# Youth Willingness to Purchase Whole Grain Snack Packs from New York City Corner Stores Participating in a Healthy Retail Program

**DOI:** 10.3390/ijerph16183233

**Published:** 2019-09-04

**Authors:** Tashara M. Leak, Felicia Setiono, Navika Gangrade, Erika Mudrak

**Affiliations:** 1 Division of Nutritional Sciences, Cornell University, 416 Savage Hall, Ithaca, NY 14853, USA; 2 Cornell Statistical Consulting Unit, Cornell University, Academic Surge A, Room 102, Ithaca, NY 14853, USA

**Keywords:** youth, whole grains, snack, low-income, urban, corner store

## Abstract

Corners stores in low-income communities are a promising setting to intervene in youth whole grain intake. One strategy that may encourage whole grain intake is if corner stores were to pair and sell whole grain snacks in combination with either a liked fruit or vegetable and an optional condiment (i.e., a whole grain snack pack). This study examined youth in terms of their (1) liking of fruits, vegetables, and whole grain snacks; (2) perceptions about which fruits and vegetables pair best with whole grain snacks; and (3) willingness to pay for a whole grain snack pack. One-time intercept surveys were conducted with 10–18-year-olds (*n* = 402) who visited a New York City (NYC) corner store (*n* = 34) participating in the City Harvest Healthy Retail Program. On average, youth were willing to spend $2.38 (SD $4.32) on a whole grain snack pack. Higher overall liking scores for vegetables and whole grain snacks were associated with willingness to spend 24.4% (95% confidence interval (CI): 11.5–38.7%) and 21.6% (95%CI: 5.2–40.6%) more on whole grain snack packs, respectively. In conclusion, youth are receptive to purchasing whole grain snack packs from NYC corner stores participating in a healthy retail program.

## 1. Introduction

Research suggests that whole grain intake is inversely associated with various health outcomes such as obesity, type 2 diabetes, and other chronic diseases [1,2,3,4]. It is important to establish healthy dietary behaviors, such as eating whole grains, during childhood and adolescence given that these behaviors often transcend into adulthood [5,6,7]. The Dietary Guidelines for Americans recommend that individuals consume at least three ounce-equivalents of whole grains per day, based on a 2000-calorie diet [8]. When examining nationally representative data, several studies have reported that U.S. children and adolescents consume less than one ounce-equivalent of whole grains on a given day [9,10,11]. Furthermore, there is evidence that youth residing in low-income households consume even fewer whole grains on a given day than their counterparts from higher socioeconomic backgrounds [10,12]. Also, a smaller proportion of racial/ethnic minority youth meet recommendations for whole grain consumption compared to those who identify as non-Hispanic White [13].

A promising setting to intervene in child and adolescent whole grain intake are corner stores. Studies have found that youth in low-income, urban communities frequent corner stores to purchase snacks [14,15]. Compared to higher income neighborhoods, impoverished urban communities have more corner stores, which often stock items with little nutritional value [16,17]. To address the limited availability and accessibility of healthy foods in corner stores, several organizations in large cities have created healthy retail programs where participating corner stores receive resources and support in order to increase the availability, accessibility, and purchase of fresh produce [18,19]. Such efforts include in-store signage, promotional giveaways, store owner training on handling fresh produce, and the improvement of store infrastructure to stock produce. To our knowledge, there have been no documented attempts to increase the purchase of whole grain snacks in corner stores participating in a healthy retail program.

Research informed by behavioral economics principles shows that pairing a less known/less liked food with a liked food [20] and/or a condiment [21] is an effective strategy to increase intake of the less known/less liked food. For example, one study found that when raw vegetables (carrots, celery, and broccoli) were served with peanut butter, middle school aged youth ate more of the raw vegetables compared to when children were served the raw vegetables alone [22]. Thus, pairing and selling whole grain snacks with a liked fruit or vegetable, and optionally a condiment may encourage youth to purchase and consume more whole grain snacks. The research team calls this proposed new combination of foods a “whole grain snack pack”. For the current study, youth visiting a corner store participating in a healthy retail program were asked to complete a one-time intercept survey in order to examine factors that may influence their willingness to purchase whole grain snack packs. Prior to working with corner stores to prepare and sell whole grain snack packs within the store, it is important to understand (1) which fruits, vegetables and whole grain snacks youth like; (2) perceptions about which fruits and vegetables pair well with whole grain snacks; and (3) how much youth would be willing to pay for a whole grain snack pack. 

## 2. Materials and Methods 

### 2.1. Recruitment and Study Design 

The current study was a collaboration with New York City (NYC) corner stores participating in the City Harvest Healthy Retail Program (*n* = 34). City Harvest is a food recovery organization, and the Healthy Retail Program provides support to participating corner stores in an effort to increase the availability and accessibility of fresh produce [23]. Corner stores enrolled in a healthy retail initiative were recruited because they have demonstrated a commitment to increasing the sales of fresh produce and thus may be motivated to also increase whole grain snack purchases by preparing and selling whole grain snack packs at their respective stores. 

In May and June of 2018, during the NYC school year, each of the 34 corner stores were visited once during the weekday (Monday–Friday) from 2–6 pm (i.e., after school hours). On the day of the store visit, two members of the research team (1) conducted a store audit to examine the availability of vegetables, fruits, whole grain snacks, and condiments that could be paired together and sold as a whole grain snack pack; (2) surveyed a corner store representative (who was at least 18 years old and could speak English or Spanish) to examine perceived facilitators and barriers to preparing and selling whole grain snack packs at the store; and (3) conducted intercept surveys with youth (10–18 years of age) exiting the store to gauge their willingness to purchase a whole grain snack pack. Note that youth were not required to have purchased anything from the corner store, but they did need to have entered the corner store in order to participate in the study.

The Cornell University Institutional Review Board (IRB) approved the study. According to the IRB, parental consent was not required, but youth were required to provide oral assent in order to participate in the study. Findings from the store audit and surveys with corner store representatives will be published elsewhere, and this manuscript focuses on the findings from the intercept survey with youth. 

### 2.2. Data Collection

The youth intercept survey was delivered via Qualtrics (Qualtrics, Provo, UT) on an iPad. After the participant provided oral assent, the researcher entered a unique ID for the borough in NYC where the corner store was located (Brooklyn, Staten Island, Manhattan, or the Bronx), a unique ID for the corner store, and a unique ID for the participant. Then, the survey began by asking about the following: participant’s age, what grade they were in for the 2017–2018 school year (grades 6–12; I did not attend school during the 2017–2018 school year; other), current gender identity (female/woman; male/man; other; prefer not to answer), and the ethnic and/or racial group that they identify with (Hispanic, Latino, or Spanish; American Indian or Alaskan Native; Asian; Black or African American; Native Hawaiian or Other Pacific Islander; White; other; prefer not to answer). 

Afterwards, youth were asked if they had ever (yes or no) eaten a variety of fruits (apples, bananas, oranges, and grapes), vegetables (carrots, cucumber, celery, broccoli, and tomatoes), and whole grain snacks (pretzels, granola bars, popcorn, cereal, and crackers). The specific food items in this survey were selected from a corner store audit tool developed by the research team. The audit tool was primarily derived from the CX3 and the Nutrition Environment Measures Survey for Corner Stores (NEMS-CS), a validated tool to measure the overall nutrition environment of retail stores [24]. Additional vegetables and fruits were added to the audit tool based on the Harvest Chart for New York City [25], while additional whole grain items were added based on other studies examining whole grain snacks [26,27]. The final food items included in the youth intercept survey were selected because they were widely available snack foods, could be eaten raw (for fruits and vegetables), and not as subject to seasonal availability. If they selected yes, they were asked to rate their liking of each of the aforementioned items using a 5-point labeled hedonic scale (dislike a lot, dislike, neither like nor dislike, like, like a lot). In addition to rating their liking of a variety of fruits, vegetables, and whole grain snacks, youth were asked to select one vegetable and one fruit that pairs best with each of the previously mentioned whole grain snack options. For example, participants were asked, “which vegetable pairs best with pretzels?” and they could select one vegetable from the list of options (carrots, cucumber, celery, broccoli, or tomatoes) or none. Also, participants were asked, “which fruit pairs best with pretzels?” and they could select one fruit from the list of options (apples, bananas, oranges, and grapes) or none. 

Lastly, a one-and-one-half-bound dichotomous choice approach was used in order to evaluate youth willingness to pay for a whole grain snack pack [28]. Youth were asked, “if a whole grain snack pack cost $0.50, would you purchase it?” (yes or no). If the participant said yes, they were then asked, “if a whole grain snack pack cost $1.00, would you purchase it?” (yes or no). This question was asked in $0.50 increments starting from $0.50 up to $5.00. This $0.50 increment was used to mimic a realistic pricing structure in a corner store. If a participant indicated that they would purchase a whole grain snack pack that cost $5.00, they were then asked, “what is the greatest amount of money that you are willing to spend on a whole grain snack pack that includes a whole grain snack and a fresh fruit or vegetable?” and this was an open-ended question.

Study participants completed the intercept survey in 5 to 10 minutes. In exchange for their time, they received a $5.00 cash incentive after completing the survey. 

### 2.3. Data Management

Each NYC borough, corner store, and participant were assigned a unique ID, all of which were entered into Qualtrics before the participant began the survey. No personal identifiers were collected. The Qualtrics survey and data were password-protected and saved on a secured shared drive. Nine study participants completed the intercept survey on paper, and their data were entered into Qualtrics. The paper surveys were stored in a folder and kept in a locked cabinet. All of the research team completed the Collaborative Institutional Training Initiative (CITI) Human Subject Research training on how to ethically collect and store data [29]. 

### 2.4. Data Analysis

Sociodemographic variables, youth liking of food items (fruits, vegetables, and whole grain snacks), and youth perceptions about fruits and vegetables that pair well with whole grain snacks were summarized using frequency and percentages (categorical variables) and using mean and standard deviations (continuous variables).

Study participants reported their liking of a variety of fruits, vegetables and whole grain snacks using a 5-point labeled hedonic scale (scored 1 = dislike a lot, scored 2 = dislike, scored 3 = neither like nor dislike, scored 4 = like, scored 5 = like a lot). Pictures of whole grain snacks on the survey were either common brands of the product (e.g., Nutrigrain Granola Bars, Nature Valley Oats n’ Honey Granola Bars, Wheat Thins, Triscuits, or brandless stock photos). Difference in liking ratings between food items within each category (i.e., comparing each fruit to one another) were examined using the Kruskal–Wallis test, which is suitable to test differences when variables are ordinal in nature [30]. Post hoc analysis with Bonferroni correction was done using the Dunn test [31]. The liking scores of fruits, vegetables, and whole grain items were assessed for collinearity. 

The proportions of how frequently each fruit (apples, bananas, grapes, oranges) and each vegetable (carrots, cucumber, celery, tomatoes, broccoli) paired best with each whole grain item (pretzel, snack bar, popcorn, cereal, cracker) were calculated. These proportions were compared to each other using two-tailed proportion tests. The proportion of youth who chose the ‘none’ option for the pairing question was also calculated for each of the whole grain items. A two-tailed, two-sample proportion test was then used to compare the proportions of youth who stated that no vegetables pair well with whole grain items and youth who stated that no fruits pair well with whole grain items. Finally, chi-square tests were used to evaluate the expected and observed frequency of specific vegetables or fruit to whole grain pairing.

Youth interest in whole grain snack packs was measured based on the maximum amount of money youth were willing to spend on a whole grain snack pack. This variable was treated as a continuous variable. We had youth report the amount of money they spent on foods and beverages from the corner store that day, and we used these data to predict the outliers for the maximum amount youth would spend on whole grain snack packs. The amount of money youth spent on food and beverages on the surveyed date was treated as a continuous variable by taking the median of the price interval (i.e., if the youth reported spending between $0.01–0.99, the youth’s spending was coded as $0.50). The first quartile, median, third quartile, and the interquartile range of this data was calculated. The outlier was calculated as 1.5 times the interquartile range above the third quartile of the current spending data and was equivalent to $15.50. Thus, if youth reported more than $15.50 when asked how much they would spend on a whole grain snack pack, their data was viewed as an outlier and excluded from the analyses (*n* = 5). 

The maximum amounts of money youth would spend on whole grain snack packs were log transformed. A value of $0.36 was added to all data points before log transformation. This was done so that those who were not willing to purchase whole grain snack packs (i.e., the maximum amount of money was equal to $0.00) had a data value of $0.36 instead of $0.00 since it is impossible to log transform values of ‘0’. A value of $0.36 was used based on the methods described by McCune and Grace 2002 [32]. Further testing indicated that adding $0.36 to the data preserves the original orders of magnitude in the data. 

Multiple linear regression was then run to assess the relationship between the maximum amount of money youth would be willing to spend on whole grain snack packs and the average liking scores of fruits, vegetables, and whole grain snacks. A natural log transformation on the maximum amount of money youth would spend on whole grain snack packs was used to meet the assumption of normally distributed residuals. 

The borough where the corner store was located, as well as self-reported age, gender, and ethnicity/race were included in the model as potential confounders. All statistical analyses were conducted in STATA Statistical Software: Release 15 (College Station, TX: StataCorp LLC, 2017). Statistical significance was set at *p* < 0.05.

## 3. Results

Corner stores were located in Brooklyn (*n* = 8), Staten Island (*n* = 6), Manhattan (*n* = 10), and the Bronx (*n* = 10). No store located in Queens agreed to participate in the study; there was only one store in Queens participating in the City Harvest Retail Program, and the owner was often not present at the corner store, which meant they were unable to be recruited to participate in the program. The 34 corner stores were located in neighborhoods with at least 15% of residents below the NYC poverty threshold ($32,402 for a two-adult, two-child family in 2016), with the majority of stores in areas with a poverty rate greater than 20% [33]. 

### 3.1. Participant Characteristics

Sociodemographic characteristics of youth (*n* = 402) who participated in the study are described in Table 1. Slightly less than half of the participants were surveyed at a corner store located in the Bronx. On average, youth were about 14 years old, with about half of the participants in grades 6–8. The percentage of youth that self-identified as male was 60%. Concerning ethnicity/race, 46% of youth self-identified as Hispanic, 35% as Black, and 11% as multi-racial. 

### 3.2. Youth Liking of Fruits, Vegetables, and Whole Grain Snacks

Average liking ratings for specific fruits, vegetables, and whole grain snacks can be found in Table 2. Oranges and grapes were significantly more preferred to apples and bananas (all *p* < 0.05, Kruskall–Wallis dominance test). Among the vegetables, carrots and tomatoes were significantly less preferred than cucumbers and broccoli (all *p* < 0.01). However, all of the vegetables were significantly more preferred than celery (all *p* < 0.01). Granola bars and popcorn were significantly more preferred compared to pretzels, cereals, and crackers (all *p* < 0.01). 

The overall median liking score for all fruits was higher than the overall median liking score for all whole grain snacks and the overall median liking score for all vegetables (*p* < 0.01). Also, the overall median liking score for all whole grains snacks was significantly higher than the overall median liking score for vegetables (*p* < 0.01).

### 3.3. Fruits and Vegetables that Pair Best with Whole Grain Snacks

The frequency and proportions (pr) of surveyed youth selecting fruits and vegetables that they felt pair best with whole grain snacks are reported in Table 3.

Among the fruits, apples (pr: 0.35) were chosen significantly more than any other fruits as pairing best with all whole grain items (all *p* < 0.0001). Bananas (pr: 0.29) were chosen significantly more than grapes (pr: 0.22) and oranges as pairing best with all whole grain items (pr: 0.14) (all *p* < 0.0001). Grapes were also chosen significantly more than oranges to pair best with whole grain items (*p* < 0.0001). 

Carrots (pr: 0.34) were cited as pairing better with all of the whole grain items than any of the other vegetables (*p* < 0.0001). Celery (pr: 0.20) was said to pair better with all of the whole grain items than cucumbers (pr: 0.16), tomatoes (pr: 0.17), and broccoli (pr: 0.13) (*p* values 0.0002, 0.0024, and < 0.0001 respectively). Lastly, cucumber and tomatoes were said to pair better with all of the whole grain items than broccoli (*p* = 0.0081 and *p* = 0.0002 respectively).

For each whole grain item, there is a significantly higher proportion of youth who indicated no vegetables pair best with the whole grain item compared to the proportion of youth who indicated that no fruits pair best with whole grain items (all *p* < 0.0001).

### 3.4. Youth Willingness to Purchase Whole Grain Snack Packs

On average, youth were willing to spend $2.38 (SD $4.32) on a whole grain snack pack. After adjustment for age, race and ethnicity, borough, and gender, we observed a significant positive association between liking scores of vegetables and whole grain items and the maximum amount of money youth were willing to spend on whole grain snack packs (*p* < 0.0001 and *p* = 0.008, respectively). On average in Table 4, those with a higher liking score for vegetables were willing to spend 24.4% more on whole grain snack packs (95%CI: 11.5–38.7%). Those with a higher score for whole grain items were willing to spend on average 21.6% more on whole grain snack packs (95%CI: 5.2–40.6%). 

## 4. Discussion

The aim of this study was to examine youth willingness to purchase whole grain snack packs from NYC corner stores participating in a healthy retail program. U.S. children and adolescents are not meeting dietary recommendations for whole grain intake [9,10,11]. Thus innovative strategies are needed to encourage an increase in consumption of whole grain. Selling whole grain snack packs may be an effective strategy to improve whole grain intake, as well as the intake of other healthy foods [20,21,22] which are shown to be associated with positive health outcomes [1,2,3,4]. Among children and adolescents who eat whole grains, a significant percentage of whole grains are consumed during snacking eating instances [9], and this is therefore a promising eating occasion in which to intervene. 

In order for corner store owners to prepare and sell whole grain snack packs, it is important to determine which fruits, vegetables, and whole grain snacks their customers like. The youth that participated in the current study generally liked all of the fruits, vegetables, and whole grain snacks listed on the intercept survey. Youth reported liking oranges and grapes more than apples and bananas. Similar to other studies [34,35], youth preferred fruits to vegetables. We found that youth liked cucumbers and broccoli more than carrots and tomatoes, but a cross-sectional examination of vegetable liking among racially/ethnically diverse 9–12-year-olds residing in a low-income household in the Minneapolis/Saint Paul, Minnesota area reported that participants had higher liking ratings for carrots and tomatoes than cucumbers and broccoli [36]. Most of what is known about whole grain liking relates to foods commonly consumed during mealtimes (e.g., pancakes, tortillas, pizza, etc.) [37,38]. Thus, the current study contributes to our limited knowledge about whole grain snack preferences among U.S. youth. Youth generally liked all five whole grain snacks included in the intercept survey, with a higher preference for granola bars and popcorn. While few studies have examined child and adolescent whole grain liking, researchers who have examined sources of whole grain intake among a nationally representative sample have found that salty grain snacks (e.g., popcorn) account for a significant proportion of childhood and adolescent whole grain intake [12,27]. 

While youth generally provided high liking ratings for whole grain snacks, pairing them with a liked fruit or vegetable may be an effective strategy to increase the purchase and intake of whole grain snacks. There is limited research examining the behavioral economics principal of pairing in relation to whole grain liking and intake, but several studies have reported that this is an effective strategy to increase vegetable intake [20,21,22]. The current study found that among the listed fruits and vegetables, apples and carrots were reported to pair best with all of the whole grain snacks. Additional research is needed on what condiments pair well with whole grains. Research conducted with Mexican–American middle school aged youth found that pairing vegetables with peanut butter led to an increase in vegetable consumption [22]. Thus, it may be worth exploring whether peanut butter pairs well with whole grains.

Determining how much youth are willing to spend on a whole grain snack pack is essential. On average, youth said that they would pay $2.38. Findings regarding how much money youth spend when visiting a corner store is varied. For example, Dennisuk et al. (2011) surveyed 242 African American youth (10–14 years) in Baltimore, Maryland and found that youth spend $3.96 on average on food and beverages a day and that corner stores were the most common place where these foods and beverages were purchased [39]. Another study reported that racially/ethnically diverse children (grades 4–6) attending a school located in a low-income, urban community spend approximately $2.00 each time that they visit a corner store [40]. Intercept surveys (*n* = 833) conducted with fourth to sixth grade students in Philadelphia, Pennsylvania revealed that youth spent $1.07 per each corner store visit on average [14]. Thus, cost is an important factor to consider before selling whole grain snack packs. 

Additionally, in the present study, liking scores for whole grain snacks and vegetables were positively associated with how much money youth were willing to spend on a whole grain snack pack. Several studies have examined this association between youth snack preferences and purchase behaviors [41,42,43]. Heard and colleagues (2016) performed an online grocery store simulation with youth and discovered that liking was associated with the increased selection of snacks, such as carrots, raisins, and apples [42]. In another study of Norwegian adolescents, liking of snack foods was correlated with willingness to purchase them [41]. However, to our knowledge, no studies have examined the relationship between youth snack liking and purchasing behaviors using a quantitative valuation method (i.e., dollar amounts quantifying willingness-to-pay). A further exploration of children as consumers of snack foods is key, as 50% of children’s first solo purchases are at a convenience store [44], and snacks are considered “normal goods” for children to spend their money on (versus luxury goods such as clothes) [45]. 

This study is not without limitations. One potential limitation is social desirability bias, as youth may have allowed the presence of research staff administering the intercept survey to influence their responses. Studies have concluded that social desirability may be a source of reporting error in nutrition and health-behavior research, specifically among children and youth [46,47]. The inclusion of measures of social desirability and response rate could have provided additional information to utilize during analysis. In this study, no dietary intake data was collected. Thus, we were unable to determine whether the study sample had representative fruit, vegetable, and whole grain consumption scores compared to the national average, which might impact the overall liking scores of fruits, vegetables, and/or whole grain snacks. Additionally, the study examined youth aged 10–18 years old in NYC, and the findings may not be generalizable to other age groups and settings, especially as NYC has an abundance of corner stores (more than 10,000) [48] that residents visit more frequently than those in other urban areas [49]. Furthermore, youth intercept surveys were only collected during after-school hours on weekdays, which may have influenced the data collected, as other studies reveal that the time of day and day of the week (weekday versus vacation/weekend) affect snacking frequency, types of snacks eaten, and the energy density of snacks eaten [50]. However, an analysis of NHANES data revealed that most children snack in the afternoon, when the present study’s youth intercept surveys were conducted [50]. Youth who were willing to participate in the survey may be a select group, those who were more interested in fruits, vegetables, and whole grains snacks, which could bias the results. However, the explanation of the survey during the recruitment of the youth was kept simple to reduce sampling bias. Youth were only asked if they would be willing to share their opinion on whole grain snack packs. This explanation was accompanied with a picture of a whole grain snack pack. Lastly, youth intercept surveys were only conducted at corner stores participating in a healthy retail program and, as such, attitudes may not be similar in those youth that frequent corner stores not participating in such programs. For these youth, certain foods on the intercept survey may not be available or accessible in corner stores without programming, and this may impact liking ratings, as liking for foods increases as exposure to them does [51]. However, it is important to note that corner stores participating in healthy retail initiatives may be more likely to introduce healthy snack options and, therefore, their youth consumers’ purchasing preferences are particularly important.

Despite these limitations, this study has several strengths. To our knowledge, this study is the first to investigate youth’s whole grain snack preferences and willingness to purchase whole grain snacks. As youth are autonomous consumers of snack foods and beverages, especially in low-income, urban settings [14,15], it is important to understand their snack purchasing behaviors and preferences. The use of intercept surveys allowed the collection of more accurate data on these behaviors and preferences, as youth may have difficulty recalling purchasing habits using retrospective methods. Furthermore, this study captured the attitudes of racial/ethnic minority youth, who have had a greater increase in number of snacks eaten per day and calories from desserts, sweets, and salty snacks compared to their White counterparts [52,53] and therefore are the target population for healthy snack options, such as whole grain snack packs. The results of this finding have been shared with participating corner stores representatives and the City Harvest Healthy Retail Program staff. Thus, future studies may focus on exploring marketing strategies to promote the sale of whole grain snack packs and test the feasibility of setting prices and selling whole grain snack packs in the participating corner stores.

## 5. Conclusions

Findings suggest that youth who frequent NYC corners stores that participate in a healthy retail program are receptive to purchasing whole grain snack packs if this product is available for purchase. It was encouraging to learn that youth like a variety of fruits, vegetables, and whole grain snacks. Corner store owners can use the information on which fruits and vegetables pair best with whole grain snacks to inform what they choose to include in a whole grain snack pack. Additional research is needed regarding which condiments may pair with a whole grain snack pack. Future studies are needed exploring the relationship between the availability of whole grain snack packs, purchasing behaviors, and dietary intake among racially/ethnically diverse youth visiting corner stores in urban communities. 

## Figures and Tables

**Table 1:** Sociodemographic characteristics of surveyed youth who visited a City Harvest Healthy Retail Program corner store during the one-time store visit (*n* = 402).

Sociodemographic Characteristics	*n* (%)
Borough	
Brooklyn	98 (24.4%)
Bronx	189 (47.0%)
Manhattan	68 (16.9%)
Staten Island	47 (11.7%)
Age ^a^, mean (SD)	13.6 (2.4)
Grade level for 2017–2018 school year? ^b^ (*n* = 401)	
6	73 (18.2%)
7	77 (19.2%)
8	56 (14.0%)
9	25 (6.2%)
10	41 (10.2%)
11	51 (12.7%)
12	30 (7.5%)
Don’t attend school	5 (1.2%)
Other ^c^	43 (10.7%)
Gender identity	
Female	157 (39.1%)
Male	240 (59.7%)
Other	3 (0.7%)
Prefer not to answer	2 (0.5%)
Ethnic and/or racial group ^d^	
Hispanic, Latino, or Spanish	184 (45.8%)
American Indian or Alaskan Native	4 (1.0%)
Asian	4 (1.0%)
Black	139 (34.6%)
Native Hawaiian or Other Pacific Islander	1 (0.2%)
White	7 (1.7%)
Multiracial ^e^	44 (10.9%)
Other	11 (2.7%)
Prefer not to answer	8 (2.0%)

^a^ Percentages are reported based on the number of responses for age (*n* = 389; i.e., missing data from three participants). ^b^ Percentages are reported based on the number of responses for grade (*n* = 401; i.e., missing data from one participant). ^c^ Other may include youth who were in the fifth grade or attending college, but still met the age inclusion criteria (i.e., 10–18 years old). ^d^ For participants who self-identified with only one ethnic and/or racial group, their data are included with the ethnic and/or racial group that they identified with. ^e^ For participants who self-identified with more than one ethnic/racial category, their data are included in the multiracial category.

**Table 2:** Average liking scores for specific fruits, vegetables, and whole grain snacks based on a 5-point labeled hedonic scale (scored 1 = dislike a lot, scored 2 = dislike, scored 3 = neither like nor dislike, scored 4 = like, scored 5 = like a lot).

Food Category	Foods (*n* = Number of Youths Who had Tried the Item) ^a^	Average Liking Score (SD)
Fruits	Apples (392)	4.52 (0.74)
	Bananas (374)	4.33 (1.06)
	Grapes (391)	4.68 (0.62)
	Oranges (390)	4.66 (0.62)
		Average liking score for fruits = 4.55 (0.53)
Vegetables	Broccoli (350)	3.97 (1.13)
	Carrots (349)	3.62 (1.06)
	Celery (261)	3.23 (1.30)
	Cucumber (299)	3.99 (1.01)
	Tomatoes (320)	3.64 (1.27)
		Average liking score for vegetables = 3.71 (0.76)
Whole grain snacks	Cereal (324)	4.10 (0.95)
	Crackers (325)	4.06 (0.94)
	Granola bars (376)	4.40 (0.78)
	Popcorn (392)	4.34 (0.83)
	Pretzels (359)	4.11 (1.02)
		Average liking score for whole grain snacks = 4.21 (0.61)

^a^*n* varies per item because the liking question was asked only to youth who had previously tried the item.

**Table 3:** The frequency and proportion of surveyed youth who visited a City Harvest Healthy Retail Program corner store during the one-time store visit who indicated which vegetables and fruits, if any, paired best with whole grain snacks.

		Cereal	Crackers	Granola Bar	Popcorn	Pretzels	Total (Proportion) ^a^
Fruits		N = 401	N = 400	N = 402	N = 401	N = 401	
	Apples	96	125	131	80	116	548 (0.35)
	Bananas	153	96	99	36	62	446 (0.29)
	Grapes	46	55	77	74	84	336 (0.22)
	Oranges	43	41	43	54	39	220 (0.14)
	None ^b^	63	83	52	157	100	
Vegetables		N = 401	N = 401	N = 401	N = 400	N = 400	
	Broccoli	24	31	33	31	28	147 (0.13)
	Carrots	64	92	79	56	94	385 (0.34)
	Celery	39	48	51	33	56	227 (0.20)
	Cucumber	33	41	43	26	34	177 (0.16)
	Tomatoes	33	53	42	34	27	189 (0.19)
	None ^c^	208	136	153	220	161	

^a^ Signifies the total number of times for which a particular fruit or vegetable was said to have paired best for whole grain snacks. Proportions were calculated from the number of times any fruit or vegetable was chosen (i.e., not none) and were calculated for fruits and vegetables separately. ^b^ The number of youths who said that none of the listed fruits paired best with a particular whole grain snack. ^c^ The number of youths who said that none of the listed vegetables paired best with a particular whole grain snack.

**Table 4:** Association between average liking scores of youth for fruits, vegetables, and whole grain snacks and the maximum cost they were willing to spend on whole grain snack packs (n=373).

Food Categories	Percentage Increase in Maximum Cost Youth Were Willing to Spend on Whole Grain Snack Packs Associated with an Increase of 1 Point in Liking Scores of Food Categories (95% Confidence Interval)
Fruits	17.3 (−0.8, 38.7)
Vegetables	24.4 (11.5, 38.8) ^a^
Whole grain snacks	21.6 (5.2, 40.6) ^a^

^a^ Statistically significant at *p* ≤ 0.05.
